# Toward Long-Term Communication With the Brain in the Blind by Intracortical Stimulation: Challenges and Future Prospects

**DOI:** 10.3389/fnins.2020.00681

**Published:** 2020-08-11

**Authors:** Eduardo Fernández, Arantxa Alfaro, Pablo González-López

**Affiliations:** ^1^Institute of Bioengineering, Universidad Miguel Hernández, Elche, Spain; ^2^Center for Biomedical Research in the Network in Bioengineering, Biomaterials and Nanomedicine (CIBER-BBN), Madrid, Spain; ^3^John A. Moran Eye Center, University of Utah, Salt Lake City, UT, United States; ^4^Hospital Vega Baja, Orihuela, Spain; ^5^Hospital General Universitario de Alicante, Alicante, Spain

**Keywords:** visual prostheses, blindness, biocompatibility, biotolerability, neuroplasticity, visual cortex

## Abstract

The restoration of a useful visual sense in a profoundly blind person by direct electrical stimulation of the visual cortex has been a subject of study for many years. However, the field of cortically based sight restoration has made few advances in the last few decades, and many problems remain. In this context, the scientific and technological problems associated with safe and effective communication with the brain are very complex, and there are still many unresolved issues delaying its development. In this work, we review some of the biological and technical issues that still remain to be solved, including long-term biotolerability, the number of electrodes required to provide useful vision, and the delivery of information to the implants. Furthermore, we emphasize the possible role of the neuroplastic changes that follow vision loss in the success of this approach. We propose that increased collaborations among clinicians, basic researchers, and neural engineers will enhance our ability to send meaningful information to the brain and restore a limited but useful sense of vision to many blind individuals.

## Introduction

Visual impairment affects personal independence, reduces quality of life, and has a significant impact on the lives of those who suffer it (Bourne et al., 2017). Although some visual pathologies can be effectively treated, and there are some novel approaches to slow down the progression of several eye diseases, including gene and stem cell therapies (Higuchi et al., 2017; Artero Castro et al., 2018; Llonch et al., 2018; Benati et al., 2019; West et al., 2019), unfortunately, there are not treatments for all causes of blindness (Fernandez, 2018). Therefore, many scientists have long dreamed of the possibility of restoring vision by using neural prosthetic devices that bypass the damaged visual pathways.

The concept of artificially producing a visual sense in the blind is based on our current understanding of the structure of the mammalian visual system and the relationship between electrical stimulation of any part of the visual pathways and the resulting visual perceptions (Fernandez and Normann, 1995; Maynard, 2001). Thus, several research groups are focusing their efforts on the development of new approaches for artificial vision based on electric stimulation of the retina (Da Cruz et al., 2016; Lorach et al., 2016; Stingl et al., 2017), optic nerve (Duret et al., 2006; Lu et al., 2013; Gaillet et al., 2020), lateral geniculate nucleus (Vurro et al., 2014; Killian et al., 2016), or visual cortex (Fernandez et al., 2005; Normann et al., 2009; Kane et al., 2013; Normann and Fernandez, 2016; Fernandez, 2018; Niketeghad et al., 2019). All of these prosthetic devices work by exchanging information between the electronic devices and different types of neurons, and although most of them are still in development, they show promise of restoring vision in many forms of blindness.

At present, retinal prostheses are the most successful approach in this field, and several retinal devices have already been approved for patients with retinal dystrophies (Da Cruz et al., 2016; Stingl et al., 2017). However, the inner layers of the retina can degenerate in many retinal diseases. Consequently, a retinal prosthesis may not be useful, for example, in patients with advanced retinal degenerations, glaucoma, or optic atrophy. Therefore, there are compelling reasons for the development of other approaches able to restore a functional sense of vision bypassing the retina.

In this framework, since the neurons in the higher visual regions of the brain are usually spared from the damage to the retina and optic nerve, several researchers are trying to develop visual prostheses designed to directly stimulate the brain. Even if only a crude representation of the surrounding physical world can be evoked, a blind individual could use this artificially encoded neural information for tasks such as orientation and mobility. This functional performance has already been attained in the field of auditory prostheses. These devices have already allowed many deaf patients to hear sounds and acquire language capabilities (Merkus et al., 2014; Glennon et al., 2019), and the same hope exists in the field of neuroprosthetic devices designed for electrical stimulation of the visual cortex.

However, in spite of all the progress in materials and neuroelectronic interfaces, the scientific and technological problems associated with the long-term biocompatibility and biotolerability of cortical electrodes, together with the difficulties associated with the encoding of visual information, are very complex. Moreover, it is still unclear how to identify the ideal candidates for a cortical prosthesis (Merabet et al., 2007). Therefore, there are still many unresolved issues delaying its development. We summarize herein some of the main biological and technical issues that still remain to be fully solved, related mainly to the field of intracortical devices, and discuss some of the challenges in this highly multidisciplinary field.

## Electrodes That Interact With the Brain in the Blind: General Remarks

Otfried Foerster was the first neurosurgeon who exposed the occipital area of one cerebral hemisphere in an awake patient (under local anesthesia) and electrically stimulated it (Foerster, 1929). He found that electrical stimulation of this region of the brain induced the perception of small spots of light directly in front of the subject. These early findings, together with the studies of Wilder Penfield and co-workers in epileptic patients (Penfield and Rasmussen, 1950; Penfield and Jaspers, 1974), established the anatomical and physiological basis for the development of a cortical visual prosthesis for the blind. Later on, Giles Brindley in England (Brindley and Lewin, 1968a, b; Rushton and Brindley, 1978) and William Dobelle in the United States (Dobelle and Mladejovsky, 1974; Dobelle et al., 1976; Dobelle, 2000) showed that simultaneous stimulation of several electrodes placed on the surface of the brain allowed blind volunteers to see some predictable simple patterns, including Braille characters and letters (Bak et al., 1990; Schmidt et al., 1996). However, there were also some problems, such as the induction of epileptic seizures and the appearance of pain due to meningeal or scalp stimulation. These issues were associated with the large active surface of the electrodes, which required high electrical currents of the order of milliamps to evoke phosphenes. In addition, these large electrodes interacted with relatively large volumes of cortex (∼1 cm^3^), resulting in very low spatial resolution of the perceived phosphenes (Christie et al., 2016; Niketeghad et al., 2019). These later findings have recently been confirmed by Beauchamp et al. (2020), who implanted two different types of electrodes on the surface of the visual cortex of two blind individuals and found that when multiple electrodes were stimulated simultaneously, phosphenes fused into larger formless perceptions, making shape recognition impossible.

Cortical artificial vision did not seem feasible until we could find a way to provide a much more focal stimulation of neurons in the visual cortex (Normann et al., 1996). This led a number of investigators to develop new approaches such as smaller intracortical electrodes designed to be similar in size to the cell bodies of the neurons they are trying to stimulate and able to penetrate through the surface of the cortex (Normann et al., 1999; Troyk et al., 2003; Wise, 2005). These new microelectrodes can be located very close to the neurons they intend to stimulate, which are situated generally at 1–1.5 mm from the cortical surface, avoiding the relatively high electrical currents required by surface electrodes. Thus, we recently implanted an array of 100 penetrating electrodes (a Utah Electrode Array) in the occipital cortex of a 57-year-old person during a six-month period, and we found that stimulation thresholds to excite neurons were in the 1-100 microamp range (Fernandez et al., 2019). This is clearly two to three orders of magnitude smaller than the currents required to evoke phosphenes using surface electrodes.

**FIGURE 1:**
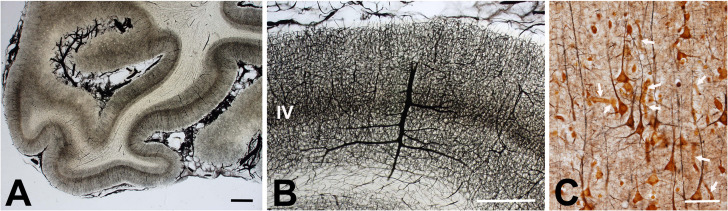
Human cerebral vascular architecture. **(A)** Section of human primary visual cortex visualized with an intravascular injection of India ink and gelatin (courtesy of Professors H. Duvernoy and P. Rabischong). Note the high density of blood vessels at the level of the gray matter. Calibration bar = 1 mm. **(B)** Detail of human gray matter vascularization showing a dense network of blood vessels at the gray matter, which is thicker at layer IV. Calibration bar = 1 mm. **(C)** Cerebral cortex impregnated with chrome-silver by Luis Simarro (image courtesy of Museum Luis Simarro, Universidad Complutense de Madrid, Madrid, Spain). Arrows indicate some blood vessels among neurons and glial cells. Calibration bar = 100 μm.

Some examples of these new penetrating neural interfaces are the arrays built with metal microelectrodes, the Utah Electrode Array, the implantable microcoils for intracortical magnetic stimulation (Lee et al., 2016), and other penetrating devices made of a variety of other materials (Fernandez and Botella, 2017). However, although these penetrating microelectrodes have been used successfully in both the central (CNS) and peripheral (PNS) nervous systems, the brain imposes some specific conditions such as the absence of regeneration and the presence of different types of glial cells. Moreover, the requirements for electrical stimulation and recording in the brain are clearly different from those in the peripheral nervous system. Thus, the brain hosts different types of neurons arranged in several superficial layers and in deep nuclei and various types of glial cells that interact in very intricate ways. Furthermore, the brain is protected by the meninges, a multi-layered structure formed by connective tissue, bone, and skin. This means that it is impossible to reach the desired cortical neurons without affecting neighboring parts of the nervous system. Likewise, the brain tissue includes a complex network of blood vessels that are likely to be injured by the introduction of any external device (Figure 1).

In addition, we should also consider the mechanical micromovements between the pulsating neural tissue (due mainly to cardiac pulse and breathing) and the static implants, which can induce different kinds of damage (Polanco et al., 2016). All of these factors place high demands on the long-term function of any intracortical electrode and also impose unique constrains for the materials, packaging, and insulation of the electronics (Normann and Fernandez, 2016).

## Biotolerability of Neural Electrodes

The implantation of any intracortical microelectrode into the brain is a traumatic procedure, and all neural electrodes to date, even those considered to be highly biocompatible, induce biological responses characterized by small microhemorrhages and a certain amount of local tissue damage around the electrodes that may impact the stability, performance, and viability of the microelectrodes. Therefore, some authors suggest that instead of biocompatibility, we should talk about biotolerability, highlighting the capacity of the microelectrodes to stay fully functional in the brain without inducing any significant tissue damage for long periods of time (Fernandez and Botella, 2017).

**FIGURE 2:**
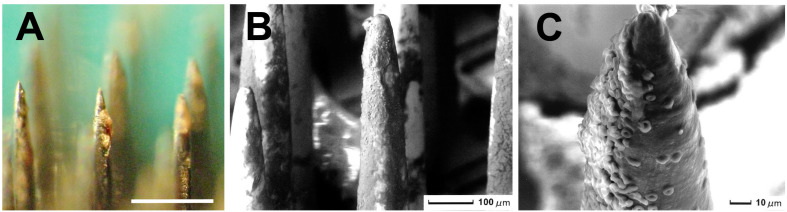
Utah Electrode Array implanted in a human brain for 10 minutes (the procedure was approved by the Ethics Committee of the Hospital General Universitario of Alicante, Spain). **(A)** Image of several electrode tips surrounded by blood cells and plasma proteins that stick to the surface of the neural electrodes. Calibration bar = 400 μm. **(B)** Scanning electron micrograph showing the surface of several microelectrodes covered by many blood cells. Calibration bar = 100 μm. **(C)** Detail of the tip of a microelectrode. Calibration bar = 10 μm.

While most materials used currently for the fabrication of intracortical electrodes remain relatively inert in the brain, they still induce a foreign-body reaction (FBR) characterized by a neuroinflammatory response of the tissue around the electrodes that may hinder the recording and stimulation of the neurons over time (Marin and Fernandez, 2010; Fernandez and Botella, 2017). Often, the FBR starts with the damage to the blood vessels encountered during the implantation of the microelectrodes in the neural tissue (see Figure 1), which causes small interstitial microhemorrhages. These microhemorrhages stop spontaneously, but there is also increased blood flow to the damaged region, together with increased permeability of local microvasculature, which induces extravasation of fluids, blood cells, and proteins toward the interstitial space. Thus, the microelectrodes become surrounded by many blood cells and plasma proteins that stick to their surface. Figure 2 shows a representative example. Therefore, blood compatibility should be considered an important issue for improving the long-term performance and viability of any neural electrode.

On the other hand, as has been reviewed in detail elsewhere (Zhong and Bellamkonda, 2008; Marin and Fernandez, 2010; Fernandez and Botella, 2017; Ferguson et al., 2019), the inflammatory responses to the implantation of any neural probe into the brain involve a large network of physiological responses including edema, release of cytokines, platelet activation, complement system activation, invasion of blood-borne macrophages, and activation of neighboring astrocytes and microglial cells (Lee et al., 2005; Polikov et al., 2005; Biran et al., 2007; Grill et al., 2009; Mcconnell et al., 2009; Marin and Fernandez, 2010). Subsequently, activated macrophages surround the microelectrodes and fuse into multi-nucleated giant cells that form a barrier, similar to a thin protective membrane, that shields brain tissue from damage (Polikov et al., 2005). Most of these processes are spontaneously resolved; however, glial scarring and giant cells can be found around many microelectrodes implanted chronically in the brain (Polikov et al., 2005). This suggests the existence of a chronic inflammation reaction that persists over time and can induce the development of a dense sheath around the microelectrodes, making it difficult to record and stimulate nearby neurons. As a result, long-term biocompatibility or biotolerability is still an unresolved issue, and most intracortical microelectrodes have a maximum *in vivo* lifetime of several months or a few years (Suner et al., 2005; Prasad et al., 2012; Barrese et al., 2013).

A significant challenge here is to reduce the neuro-inflammatory response. In recent years, several strategies for minimizing trauma and the inflammatory responses have been investigated, for example, the reduction of the cross-sectional area of the electrodes (Seymour and Kipke, 2007) and the use of more flexible and soft materials that better match the properties of the surrounding tissue (Patel et al., 2016; Fernandez and Botella, 2017; Cuttaz et al., 2019; Wang et al., 2019). However, these modifications also affect the mechanical properties of the electrodes and could result in a lack of the mechanical strength needed to withstand insertion without buckling and breaking. Another relatively simple way to control the biological responses and improve the long-term biotolerability of neural electrodes is the modification of the chemical composition of the surface of the electrodes by using different polymers and nanomaterials (Hara et al., 2016; Fernandez and Botella, 2017; Gulino et al., 2019). Moreover, we should also consider that the electronics and the connecting pathways to individual microelectrodes must be completely insulated and have to remain perfectly functional over time, which also imposes unique constraints on hermetic packaging (Jiang and Zhou, 2009; Vanhoestenberghe and Donaldson, 2013).

Although it is often not mentioned, an important issue for the long-term success of any neural implant is the quality of the surgical implantation procedures. Thus, we believe that many difficulties encountered in chronic experiments could be directly related to problems during surgery and implantation. Careful implantation seems to increase the biotolerability and long-term longevity of intracortical microelectrode arrays, and there is no way to substitute for good planning and an adequate surgical technique.

## Number of Electrodes Required for Functional Vision

The functional vision that could be restored with an array of intracortical microelectrodes implanted into the brain is a function of many parameters, but it is in part related to the number of implanted electrodes, the interelectrode spacing, and the specific location of each microelectrode in the brain (Cha et al., 1992; Dagnelie et al., 2006). However, the assumption that visual perception will improve by increasing only the number of electrodes may be incorrect.

Although we see with the brain, the input information to the visual system begins at the eye, which catches and focuses light onto the retina. The human retina is approximately 0.5 mm thick and contains both the photoreceptors or sensory neurons that respond to light and intricate neural circuits that perform the first stages of imaging processing. The output neurons of the retina are the ganglion cells, which send their axons (approximately 1–1.5 million per eye) through the optic nerve to the brain (Watson, 2014). This means that, in order to encode all the features of objects in the visual space (for example, their form, localization, contour, intensity, color, etc.) and the change of these features in time in the same way that the human retina does, we would need at least 1 million parallel channels, which is clearly well beyond the state-of-the-art of current prosthetic technologies.

Fortunately, despite the above-mentioned figures, the results of several simulation studies suggest that the amount of visual input required to perform basic visually guided tasks is not as great as one might expect. In a series of psychophysical experiments, it has been estimated that 625 electrodes implanted at the primary visual cortex could be enough for reading (although to lower speeds) and to navigate through complex visual environments (Cha et al., 1992). In this framework, the possibility of providing some degree of functional vision to facilitate the activities of daily living with only around 600–700 electrodes is very encouraging (Dagnelie et al., 2006). However, this low number of electrodes also usually implies a “tunnel vision”: a restricted visual field that can be a serious problem for orientation and mobility. To cope with this problem, we can implant several arrays of penetrating microelectrodes at different locations of the visual cortex. In this context, multiple microelectrode arrays have already been implanted in monkey visual cortex (Chen et al., 2017; Roelfsema and Holtmaat, 2018; Van Vugt et al., 2018; Self et al., 2019) and these implants are providing a better understanding of how the brain enhances the representations of visual objects in different visual regions (Klink et al., 2017; Self et al., 2019). However, more experiments are still needed, and probably the question of how many electrodes are necessary to restore a limited but useful vision will only be addressable by future experiments in blind subjects.

## Engineering a Wireless Intracortical Device With Hundreds of Electrodes

Although ongoing studies suggest that electrical stimulation via multiple electrodes may give rise to useful vision, extensive efforts are still needed to address the engineering challenges of realizing an intracortical device containing hundreds of electrodes. Furthermore, the device must be wireless, since it is necessary to avoid wires to reduce post-surgical complications such as, for example, the risk of infection. In this context, power and communication constraints, as well as power dissipation in the brain, could pose significant challenges (Sahin and Pikov, 2011; Lewis et al., 2015). Other relevant issues in this framework are the so-called “crosstalk” or interference between stimulating electrode sites and the multiplexing of stimulation channels (Barriga-Rivera et al., 2017). Thus, there is a clear need to develop new implantable technologies optimized for high channel count.

On the other hand, patients with retinal implants have to undergo long fitting procedures to measure thresholds and fine-tune the stimulation parameters on each individual electrode, but these procedures are not viable if hundreds or thousands of electrode sites need to be tested. Therefore, we need further procedures for fitting devices containing hundreds of electrodes in patients. A possible approach to facilitate the fitting procedures could be to develop bidirectional intracortical devices able to record the neuronal activity in response to electrical stimulation and use the recorded neural activity to optimize the stimulation parameters (Rotermund et al., 2019). Another possibility could be to use machine learning to find optimal stimulation settings (Kumar et al., 2016). In any case, more studies are still needed.

## Delivery of Information to Implants

Besides the number of electrodes and the engineering challenges, a key issue for the future success of cortical visual implants is related to how the brain understands artificially encoded information. All visual prostheses developed to date provide very poor vision, with relatively low spatial resolution; therefore, great efforts are still needed to design and develop new systems that can have results similarly successful as those achieved with cochlear implants.

Part of the success of cochlear implants seems to be related to the development of sophisticated signal-processing techniques and bioinspired coding strategies developed over the years (Clark, 2015; Boulet et al., 2016; Jain and Vipin Ghosh, 2018). Despite these encouraging results, most visual prosthesis devices only try to emulate the phototransducer aspects of the retina and do not consider the complex processes that are found in the mammalian visual system. Some researchers have proposed that performance could be increased significantly by incorporating neural code (Nirenberg and Pandarinath, 2012), whereas others promote the use of computer vision algorithms and techniques of artificial intelligence (Sanchez-Garcia et al., 2020). Although more studies are still needed, we expect that bio-inspired visual encoders based on intelligent signal and image-processing strategies, together with new cutting-edge artificial intelligence algorithms running neuromorphic hardware, could have a significant impact in the future to facilitate the interpretation of the processed signals (Fernandez, 2018).

On the other hand, whereas there are many relevant aspects in a visual scene (for example, form, color, and motion), most current coding strategies are only aimed at addressing the spatial details. This could be an oversimplification since, for example, the ability to recognize patterns in a scene, or the perceived receptive field size, is critical for many visual tasks. Thus, we can extract complex information, such as identifying human faces, from relatively poor-quality images by using specific cues and multiple visual features (Sinha, 2002). This suggests that besides image resolution, we should try to pay attention to other relevant visual attributes such as receptive field size, localization, orientation, or movement.

Another important issue is to focus on the specific needs of the end users. For example, some people may place more demands on object- or person-identification, whereas others could prefer to focus on orientation and mobility. The key issue is to encode and send useful information that can be translated into functional gains for daily life activities (Merabet et al., 2007). In addition, it is possible that there are subtle differences in the perceived visual field or in coding among subjects. Therefore, future advanced systems to interact with the brain in the blind should allow the customization of the functions to satisfy the particular needs and capabilities of each user.

## Neural Plasticity

The adult visual cortex does not completely lose its functional capacity after years of deprivation of visual input (Brindley and Lewin, 1968a); however, there is clear clinical evidence showing adaptive neurophysiological changes in the brain, specifically at the occipital lobe. Therefore, a relevant question is whether these adaptive changes could have a significant impact on the success of a cortical visual prosthesis.

In response to the loss of vision, brain areas normally devoted to the processing of visual information are recruited to process tactile and auditory information and even cognitive functions such as verbal memory and speech processing (Fernandez et al., 2005; Gilbert et al., 2009; Legge and Chung, 2016; Beyeler et al., 2017; Singh et al., 2018; Castaldi et al., 2020). These changes are related to the capability of blind subjects to extract greater information from other senses such as touch and hearing. Thus, neuroplasticity can be viewed as an adaptive and dynamic process able to change the processing patterns of sensory information.

This neuroplasticity implies that the brain undergoes important remodeling and adaptive changes after the onset of the blindness that could directly impact the success of any cortical prosthesis (Glennon et al., 2019). Over time, these adaptive changes may lead to the establishment of new connections and functional roles of different brain areas, which is probably influenced by factors such as the cause of the visual loss and the duration of visual deprivation. All these issues may help to define a preferred time window for improving the likelihood of success of any device intended for communicating with the brain in the blind.

On the other hand, it is unlikely that the re-introduction of the lost sensory input alone will be able to promptly restore sight. Therefore, we should try to develop specific strategies to communicate with the brain of the blind in order to increase the chances of extracting useful information from the artificially encoded stimulation. Furthermore, we should consider the challenges of visual rehabilitation. Thus, improved rehabilitation strategies after the surgical implantation could contribute greatly to ever improving the performance of the neuroprosthetic devices.

## Conclusion and Future Perspectives

The development of new prosthetic technologies for restoring vision to many blind individuals for whose impairment there is currently neither prevention nor cure is a must for the future.

Cortical prostheses based on penetrating microelectrodes show promise for restoring some limited but useful vision to subjects with certain forms of blindness, but the scientific and technological problems associated with safe and effective communication with the visual brain are very complex, and there are still many unresolved issues delaying its development. We expect that ongoing research on the interactions between intracortical microelectrodes and the local cellular environments, along with a better understanding of neuroplasticity and progress in medical technologies, materials science, neuroelectronic interfaces, neuroscience, and artificial intelligence, will allow advances toward the success envisioned by this technology. Nevertheless, we should go step by step and not create false expectations or underrate the challenges that still remain to be resolved. In this framework, we propose that increased collaborations among clinicians, basic researchers, and neural engineers will enhance our ability to send meaningful information to the visually deprived brain and will help to restore a limited but useful sense of vision to many profoundly blind people.

## Data Availability Statement

The datasets generated for this study are available on request to the corresponding author.

## Ethics Statement

The studies involving human participants were reviewed and approved by the Hospital General Universitario de Alicante. The patients/participants provided their written informed consent to participate in this study.

## Author Contributions

EF, AA, and PG-L contributed to the design and implementation of the research and writing of the manuscript. All authors contributed to the article and approved the submitted version.

## Conflict of Interest

The authors declare that the research was conducted in the absence of any commercial or financial relationships that could be construed as a potential conflict of interest.
